# Revealing the impact of temperature in battery electrolytes via wavelength-resolved neutron imaging

**DOI:** 10.1126/sciadv.adi0586

**Published:** 2023-09-29

**Authors:** Eric Ricardo Carreon Ruiz, Jongmin Lee, Markus Strobl, Natalie Stalder, Genoveva Burca, Lorenz Gubler, Pierre Boillat

**Affiliations:** ^1^ Electrochemistry Laboratory (LEC), Paul Scherrer Institut (PSI), 5232 Villigen PSI, Switzerland.; ^2^ Laboratory for Neutron Scattering and Imaging (LNS), Paul Scherrer Institut (PSI), 5232 Villigen PSI, Switzerland.; ^3^ Niels Bohr Institute, University of Copenhagen, Nørregade 10, 1165 Copenhagen, Denmark.; ^4^ STFC-Rutherford Appleton Laboratory, ISIS Facility, Harwell OX11 0QX, UK.; ^5^ Faculty of Science and Engineering, The University of Manchester, Alan Turing Building, Oxford Road, Manchester M13 9PL, UK.; ^6^ Diamond Light Source, Harwell Science and Innovation Campus, Fermi Ave, Didcot OX11 0DE, UK.

## Abstract

Understanding the limitations of electrolyte mixtures under extreme conditions is key to ensure reliable and safe battery performance. Among advanced characterization methods, time-of-flight neutron imaging (ToF-NI) is unique for its capability to map physicochemical changes of H-containing materials inside metallic casings and battery packs. The technique, however, requires long exposures in pulsed sources, which limits its applicability, particularly for analysis at low temperatures. To overcome these limitations, we use high–duty cycle ToF-NI at a continuous source, demonstrating its capability to expose physical and chemical changes of electrolytes due to variations in the overall molecular diffusion. The strategy described in this work reduces the exposure required and provides the baseline to study the thermal stability of electrolyte mixtures, from the proofing of state-of-the-art electrolyte mixtures up to their performance in batteries. This analysis and methodology apply to hydrogenous materials well beyond electrolytes for a wide range of applications.

## Teaser

High–duty cycle ToF-NI is used to provide a baseline for thermal stability of electrolyte mixtures and reduce exposure.

## INTRODUCTION

Developing stable lithium-ion batteries (LIBs) is crucial to ensure high performance and safety, particularly in portable and automotive applications (*1*, *2*). The low-temperature (LT) operation and increase in charging rate impose extreme conditions on battery materials resulting in a detrimental cycle of performance loss, fast charge, and fast degradation (*3*–*6*). Temperature variations, i.e., ambient conditions and fast charging, may result in partial solidification and the dissolution of electrolyte components affecting lifetime, conductivity, and transport capabilities (*3*, *7*–*9*). Development of stable electrolytes (*10*–*13*) must be accompanied by understanding their limitations in LIBs through advanced noninvasive methods sensitive to composition, molecular motions, and physicochemical changes arising from operation at subfreezing temperatures. Electrolyte characterization techniques, including Raman spectroscopy, nuclear magnetic resonance, and Fourier-transform infrared spectroscopies (*14*–*17*), require complicated procedures to extract the electrolytes or specific battery designs, hindering the true performance analysis of electrolyte mixtures, especially at LTs (*12*).

In recent years, neutron imaging (NI) methods, particularly wavelength-resolved NI, have excelled in characterizing electrochemical systems, including fuel cells (*18*, *19*) and batteries (*20*–*22*), due to their sensitivity to light elements, i.e., H and Li. By combining neutron spectral and imaging methods, such as in time-of-flight NI (ToF-NI), we demonstrated the capability of the neutron microscopic cross sections, σ*_T_*(λ), to detect functional groups and overall dynamics changes as a result of the motions (molecular vibrations or molecular diffusion) of H atoms (*23*). Traditionally, ToF-NI is used with a pulsed beam having a low duty cycle, to reach sufficient wavelength resolution to resolve features such as Bragg edges (*24*). Unless the experiments are performed at a pulsed source (a constraint that limits their range of application), pulsing the beam results in a substantial loss of average neutron flux and, in consequence, long exposures incompatible with practical electrochemical in situ studies. However, the neutron attenuation spectrum of H-based molecules does not include sharp features, and only a limited wavelength resolution is required for their analysis. By using choppers with a high duty cycle, we can therefore perform measurements at continuous beam lines with a moderate reduction of the average neutron flux. Such an approach will also be applicable to the Optical and Diffraction Imaging with Nueutrons (ODIN) instrument at the new European Spallation Source (ESS) (*25*), which will feature a duty cycle as high as 10%.

In this work, we demonstrate how high–duty cycle ToF-NI can be used to characterize the thermal stability of electrolytes in subfreezing conditions and to investigate commercial batteries in situ. We show the correlation between spatially resolved contrast variations and changes in the atomic motion of H for visualizing and quantifying physical (phase transitions) and chemical (concentration variation) changes. Using high–duty cycle ToF-NI aids in the understanding of the temperature-dependent behavior of electrolytes and batteries and is expected to assist in the development of suitable mixtures and additives to improve thermal stability and prevent battery degradation.

## RESULTS AND DISCUSSION

### Temperature-dependent nature of neutron cross sections

**Fig. 1.**
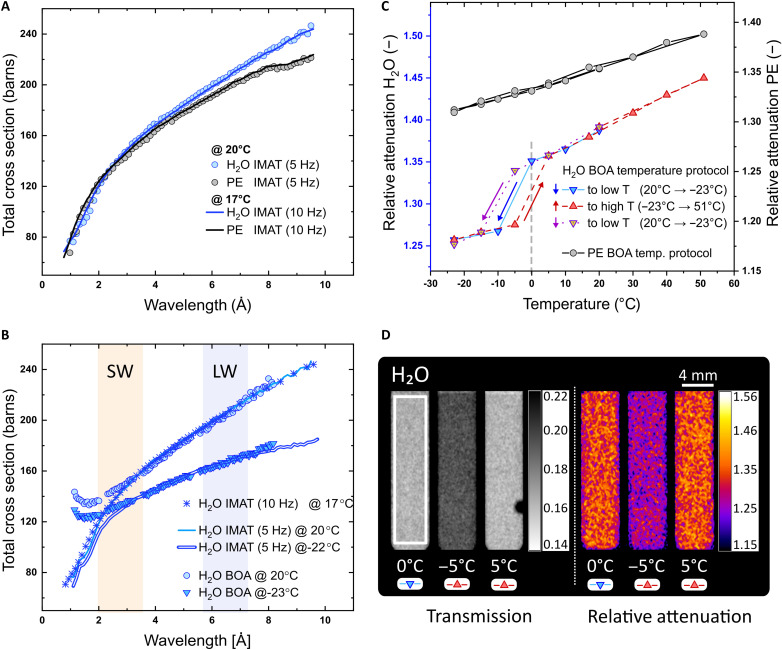
Temperature dependence of σ*_T_*(λ) and α_rel_ hysteresis. (**A**) σ*_T_*(λ) curves of H_2_O and PE measured at the IMAT. The 5-Hz repetition rate includes the temperature-controlled setup (sample-to-detector distance of 5.63 ± 0.5 cm) with 1-hour exposure (5-min ToF acquisitions). The 10-Hz repetition rate includes only the sample holder, i.e., without the temperature-controlled setup (sample-to-detector distance of 0.8 ± 0.05 cm) with 4-hour exposure (5-min ToF acquisitions). (**B**) Comparison of the BOA and IMAT σ*_T_*(λ) for H_2_O liquid and solid phases, and the selection of SW and LW regions. (**C**) H_2_O and PE α_rel_ revealing the σ*_T_*(λ) temperature dependence. The arrows follow the thermal history of the experiment demonstrating aggregation state changes and supercooled states. (**D**) Comparison between *T*_img_ and the *r*α_img_. The white rectangle corresponds to the ROI selected on the calculation of the α_rel_, which is thickness independent. Therefore, bubbles resulting from the phase transition from solid to liquid visible on the *T*_img_ do not appear in the *r*α_img_ nor influence the α_rel_.

The ToF-NI provides spatially resolved information on battery materials inside metal casings, where variations of σ*_T_*(λ) are correlated to temperature-induced physical and chemical changes. The first measurements were performed on reference materials, H_2_O and polyethylene (PE), at the Imaging and Materials Science and Engineering (IMAT) beamline (ISIS) at room temperature (20 ± 0.5°C) and 5 Hz (Fig. 1A). Although reducing the exposure by a factor of 3 (1-hour total exposure), the precision of the σ*_T_*(λ) was comparable to the previous measurements (IMAT at 10 Hz) (*23*) and literature (*26*, *27*) with an average percentage variation of 0.32 and 1.15% for H_2_O and PE, respectively. The reduction of exposure time, however, influences the relative noise as a function of the total flux $\left(1/\sqrt{\mathrm{flux}}\right)$. Hence, the total exposure time was determined to keep a statistical variation below the systematic variations (2%) with our setup: 1 hour at the IMAT with 5-min ToF and 30 min at the Beamline for neutron Optics and other Approaches (BOA) with 5-min ToF.

Each hydrogenous material has a unique footprint (Fig. 1A) in the σ*_T_*(λ) spectrum depending on the H atoms motions for molecular vibrations (over the whole σ*_T_*(λ) spectrum) or diffusion [in the long-wavelength (LW), λ > 6 Ȧ, region; (*23*)]. Therefore, variations of temperature, composition, or aggregate state are manifested as changes on the σ*_T_*(λ) spectrum with prominent differences in the LW region. As illustrated in Fig. 1B, the neutron attenuation spectrum (using the example of liquid water at 20°C and ice at −22°C) using a high–duty cycle setup (BOA) compares well with the reference measurements at a pulsed source (IMAT) for a wavelength range of 3 to 8 Å. The deviation in the left region of the short wavelength (SW) (λ < 3 Ȧ), in the BOA curves, is attributed to the pulse overlap (*18*); slow neutrons from one pulse and fast neutrons from the next pulse arrive simultaneously at the detector. The pulse overlap effects are consistent throughout the measurements and do not influence the results in this work.

To analyze the temperature-dependent nature of the σ*_T_*(λ) for liquid and solid hydrogenous materials, we investigate the ratio between the LW and SW regions, named relative attenuation (α_rel_), as shown in Fig. 1C. Where there is no phase transition, the variation of α_rel_ is due to the change in temperature only. However, sudden shifts in the α_rel_ are observed when a phase transition takes place, e.g., the transition from solid to liquid of H_2_O, and vice versa. The ice maintains the approximately linear relation between α_rel_ and temperature but with a different slope, which is similar to that of PE. At −5°C, the value of α_rel_ from the first cycle to high temperature (HT) is different to the value of α_rel_ from the second cycle to LT. Thus, the α_rel_ provides additional information on the supercooled state of a liquid that is relevant to the thermal history.

A substantial advantage of the ToF-NI method over other spectroscopic methods is the possibility to characterize real-time changes inside metal casings based on transmission images, as shown in Fig. 1D. Processed transmission images in grayscale, *T*_img_, reveal the behavior of H_2_O due to the thermal history. For the most part, only a change of contrast is distinguished at different temperatures, but, in some cases, a group of pixels with higher gray values may appear, as in the *T*_img_ at 5°C, where a bubble was generated due to the phase transition from solid to liquid. The relative attenuation images, *r*α_img_, are thickness independent. Thus, they assist in differentiating changes in the thickness from changes in the composition as a result of temperature variations (*r*α_img_ at 5°C Fig. 1D). This demonstrates that only physical and chemical changes (solidification and change of concentration) can be visualized by the *r*α_img_ without an a priori knowledge of the material thickness.

### Phase transition and temperature dependence of pure organic solvents

**Fig. 2.**
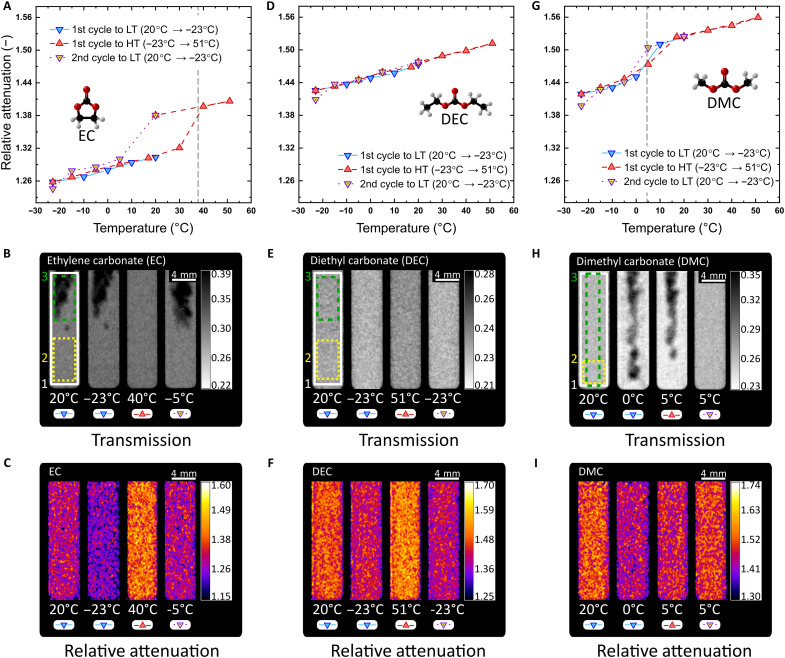
Analysis of the α_rel_ on the phase transition of pure organic solvents. (**A** to **C**) EC α_rel_ hysteresis, *T*_img_, and *r*α_img_. The dark regions in (B) are zones with a different thickness than that of the cuvette (3 mm) due to solidification. (**D** to **F**) DEC α_rel_, *T*_img_, and *r*α_img_. DEC transition temperature is lower than the range studied (−74°C). Therefore, only the σ*_T_*(λ) temperature dependence is noticed in (D) without a visible phase change in (E). (**G** to **I**) DMC α_rel_ hysteresis, *T*_img_, and *r*α_img_. The white, yellow-dotted, and green-dashed rectangles in the *T*_img_ correspond to the ROIs selected to calculate the α_rel_. The deviation among all ROIs is below 0.13%. Gray dashed lines indicate the phase transition temperature (*30*). The *r*α_img_ in (C), (F), and (I) prove the independency of α_rel_ against a change of thickness.

The three commercial electrolytes (LP30, LP40, and LP47) that are used in this work are composed of a lithiated salt and a binary mixture of ethylene carbonate (EC) and a linear carbonate, i.e., dimethyl carbonate (DMC) or diethyl carbonate (DEC). In this first part of our study, we focus on the individual components. EC is widely used in nonaqueous electrolytes due to its high dielectric constant (increase of Li-based salt solubility), low viscosity (improve Li^+^ transport), and low polarization on the cathode (*28*, *29*). EC is solid at room temperature with a relatively high melting point of around 36°C (*30*). As shown in Fig. 2A, the α_rel_ in EC begins from a low point and decreases constantly during the first cycle to LT, indicating no phase change. The solid aggregation state can be verified with the *T*_img_ (Fig. 2B), where the black (left, top right) region is caused by the crystallization of EC before the experiment and contains a lower amount of EC than that of the cuvette thickness. Figure 2C demonstrates, as with the case of gas bubbles in water in Fig. 1, that the *r*α_img_ is not affected by the thickness of the material, showing no deviation in the EC-depleted region. At 40°C, EC becomes liquid as shown by the sharp increase in the α_rel_, which continues in liquid state during the remaining points of the first cycle to HT and even back to 20°C (16°C below its freezing point), denoting a supercooled state. The recrystallization of EC is visible at 5°C in the *T*_img_ forming a hysteresis in the α_rel_. Three regions of interest (ROIs) in Fig. 2B are the areas used to calculate the α_rel_: full sample (white rectangle), bottom region with no thickness change (yellow dotted rectangle), and top region with a variant thickness (green dashed rectangle). The results presented in the α_rel_ correspond to the full sample, as the percentage deviation from the mean in all three ROIs is below 0.10%.

DEC, known to increase capacity retention and life cycle in batteries (*31*), is a pure organic solvent used in the binary mixtures of LP40 and LP47 with a solidification point around −74°C (*30*). In the temperature range of this experiment, no phase transition is expected. The α_rel_ chart in Fig. 2D shows the same constant temperature-dependent behavior without a sharp decrease indicating the liquid state of the sample throughout the experiment. The *T*_img_ and the *r*α_img_ (Fig. 2 (E and F, respectively) present only a temperature-dependent contrast change, which corroborates the assumption that there is no change of aggregation state. The percentage deviation from the mean among the ROIs shown in Fig. 2E is 0.08%.

DMC is a pure organic solvent used in the binary mixture of LP30 due to its stability at high potentials, high ionic conductivity, and high oxidative stability (*32*, *33*). Its melting point is 4.6°C, which is consistent with our measurements in Fig. 2G. A phase transition was detected in the first cycle to LT (transition between 10° and 0°C), where the sample was fully solidified (Fig. 2H) and a sharp decrease in the α_rel_ is detected. The *T*_img_ show a rather interesting crystallization of DMC from the outer part to the center of the sample (reducing the thickness at the center), different from that of EC, which generates a cavity only at the top of the cuvette. We attribute the distinctive crystallization of DMC to its low viscosity and slow solidification process during temperature decrease (EC has a high viscosity and fast solidification due to its supercooled state). DMC, as EC and DEC, presents no change in the *r*α_img_ other than a change of contrast over the whole cuvette (Fig. 2I). The percentage deviation from the mean among the ROIs is 0.13%.

A remarkable feature of the ToF-NI, in combination with the analysis of α_rel_, *T*_img_, and *r*α_img_, is that transitioning aggregation state phases can be analyzed as described in the 5°C point of the first cycle to HT. The DMC sample appears to be solid in the *T*_img_. However, the *r*α_img_ (and its quantitative α_rel_ representation) demonstrates that DMC is transitioning in the cuvette with a contrast situated between the fully liquid and fully solid phases. The total stabilization time from −5° to 5°C was 1 hour with a hold of at least 30 min at 5°C, indicating a stable liquidus phase that becomes relevant for the electrochemical and thermal stability in electrolytes, which highlights the importance of thermal history for analyzing nonaqueous electrolyte materials.

### Change of concentration in binary mixtures due to temperature

**Fig. 3.**
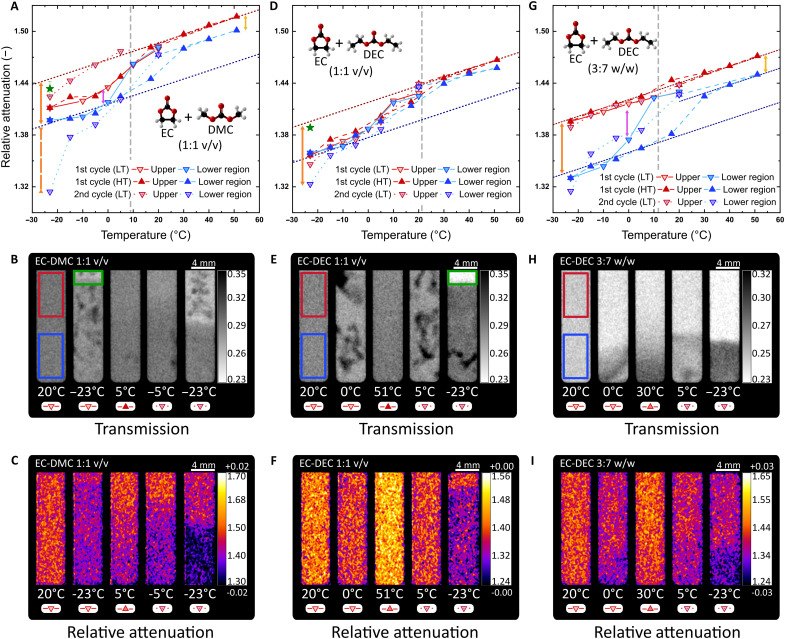
Separation of binary mixtures due to phase transition. (**A** to **C**) EC-DMC 1:1 v/v α_rel_ hysteresis, *T*_img_, and *r*α_img_. While the α_rel_ reveals the mixture separation below the transition temperature (gray dashed line), the *T*_img_ and *r*α_img_ show physical changes (solidification) and chemical changes (concentration change), respectively. (**D** to **F**) EC-DEC of 1:1 v/v α_rel_ hysteresis, *T*_img_, and *r*α_img_. Although the mixture solidifies at higher temperatures, the separation is only visible at −23°C in the second cycle to LT (green rectangle). (**G** to **I**) EC-DEC of 3:7 w/w α_rel_ hysteresis, *T*_img_, and *r*α_img_. The difference in molar fraction between EC and DEC separates after the transition temperature and creates two stable EC-enriched (bottom) and EC-depleted (top) regions. The red and blue rectangles in the *T*_img_ correspond to the ROIs selected to calculate the α_rel_ for the top and bottom regions, respectively. The green rectangles show ROIs at a given temperature noted with a green star in the α_rel_ plots. Orange arrowed lines at −23°C show the difference between the first and the second cycles to LT to emphasize the α_rel_ of a change of concentration.

In this section, we demonstrate the influence of temperature on the chemical composition of organic binary mixtures used commercially for LP30 (EC-DMC of 1:1 v/v), LP40 (EC-DEC of 1:1 v/v), and LP47 (EC-DEC of 3:7 w/w). The choice of organic solvents determines important parameters such as viscosity, salt dissociation, and electrochemical and thermal stability, which affect the overall performance of a battery (*12*, *34*). In this section, we demonstrate the influence of temperature on the chemical composition of organic binary mixtures used commercially for LP30 (EC-DMC of 1:1 v/v), LP40 (EC-DEC of 1:1 v/v), and LP47 (EC-DEC of 3:7 w/w). Furthermore, the ratio of mixtures also plays a key role, as the phase transition has a dependence on the molar fraction of each solvent (*30*, *35*). Our experiments show that exposing the mixtures below their liquidus transition temperature triggers a partial solidification, where molecules diffuse and form agglomerates with species of their own kind. For instance, Fig. 3A displays a reduction on the α_rel_ close to the liquidus transition [9°C for EC-DMC of 1:1 v/v; (*30*)]; below that point, two regions (top part and bottom part of the cuvette) with different α_rel_ are identified. Because the density of EC is greater than that of DMC, it is expected that an EC-enriched fraction is present at the bottom of the cuvette and a DMC-enriched at the top, which is the main reason why we analyzed the two parts separately. Ding *et al.* (*30*, *35*) proved that the liquidus temperature in binary mixtures varies according to the molar fraction present in the mixture. The separation of the α_rel_ during the first cycle to LT indicates the concentration change; this is also confirmed in the first cycle to HT where the stable liquid phase temperature is reached on the top (DMC-enriched) before that of the bottom (EC-enriched). The difference between the top and bottom regions remain constant throughout the HT cycle until the second cycle to LT, where the segregation between these two regions further increases, as clearly visible in both the *T*_img_ (Fig. 3B) and the *r*α_img_ (Fig. 3C). The last point (−23°C) exhibits a similar α_rel_ and solidification (top part of the *T*_img_) that of the pure DMC, while the bottom part moves toward pure EC. While *T*_img_ reveals immediate physical features (e.g., bubbles or cavities), the *r*α_img_ discerns chemical variations such as change of concentration and mixture separation.

It is important to notice that the liquidus temperature of the EC-DMC mixtures does not change monotonically with their concentration (*30*). Starting from 4.9°C for pure DMC, the liquidus temperature decreases with increasing EC fraction for values up to a molar fraction of 30% EC and 70% DMC, at which point the liquidus and solidus temperatures converge at −7.6°C. At higher fractions, the liquidus temperature increases with increasing EC fraction up to a value of 36.3°C for pure EC. The separation between an EC-enriched region at the bottom and an EC-depleted region at the top is shown both by the fact that the bottom region requires higher temperatures to fully melt than the top region and by the differences in α_rel_ remaining at the highest temperatures, where both regions are fully molten. We detected the beginning of this change of local concentration via the *T*_img_, the *r*α_img_, and its corresponding α_rel_ in the first cycle to LT at the top of the cuvette (green data point in Fig. 3A and green rectangle in Fig. 3B for both the *T*_img_ and the *r*α_img_).

The binary mixtures containing DEC (Fig. 3, D to I) present two behaviors attributed to the concentration of EC in EC-DEC of 1:1 v/v (65:35 molar fraction) and EC-DEC of 3:7 w/w (37:63 molar fraction). Higher fractions of EC have higher liquidus temperature [22°C verus 12°C, respectively; (*35*)]. However, they are less susceptible to separations at LTs (Fig. 3, D and G). The reason is the dissimilar transition temperatures of both solvents and the widespread of EC across the sample. Thus, EC-DEC of 1:1 v/v first solidifies as a unity, while EC-DEC of 3:7 w/w separates into two regions at 0°C, and the separation continues throughout the experiment. In agreement with Ding *et al.* (*35*) for the phase transition temperatures, we determined that the data points in the red dashed line (Fig. 3, D and G) are liquid mixtures of EC-DEC with an EC molar fraction close to 0.06. The green rectangle in Fig. 3E exhibits the separation to which the α_rel_ at −23°C in the top and bottom regions are similar to that of EC-DEC of 3:7 w/w (Fig. 3G) with an error of ±0.03 and ±0.62%, respectively. We demonstrate that, according to solvent fraction, the mixtures gradually separate under thermal cycles, forming agglomerates of each species and changing their concentration and physicochemical properties.

### Stability of electrolytes at LTs

**Fig. 4.**
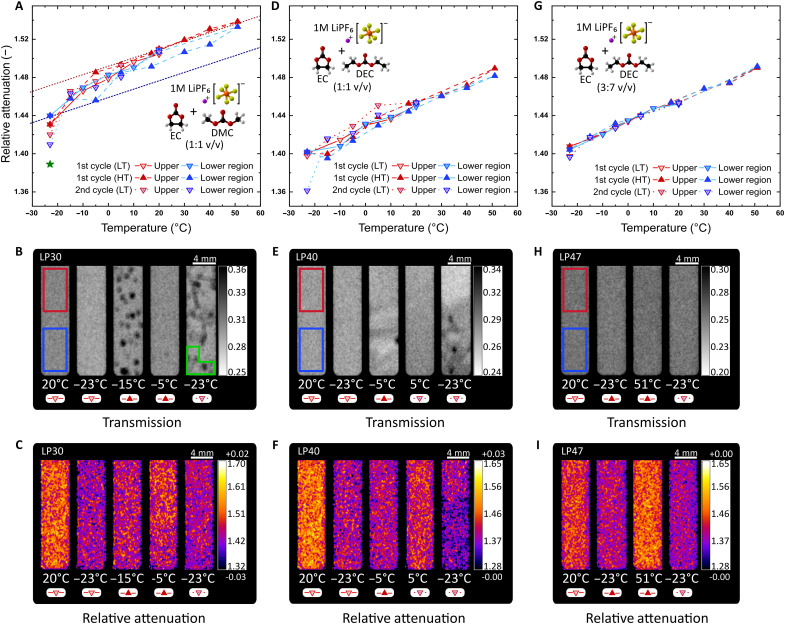
Behavior of commercial electrolytes under temperature variations. LP30, LP40, and LP47 α_rel_ hysteresis (**A**, **D**, and **G**), *T*_img_ (**B**, **E**, and **H**), and *r*α_img_ (**C**, **F**, and **I**), respectively. The addition of a lithiated salt in the binary mixtures resulted in better chemical stability under temperature variations with LP47 excelling at the studied temperature range. The red and blue rectangles in the *T*_img_ correspond to the ROIs selected to calculate the α_rel_ for the top and bottom regions, respectively. The green rectangle shows ROIs at a given temperature noted with a green star in the α_rel_ plots.

The LP30, LP40, and LP47 are Li-based aprotic electrolytes developed by adding 1 M of LiPF_6_ salt into a binary mixture: EC-DMC of 1:1 v/v, EC-DEC of 1:1 v/v, and EC-DEC of 3:7 w/w, respectively. Lithiated salts are a critical component in LiBs, as they are responsible for the ionic conductivity of the system (*36*, *37*). In contrast to binary mixtures (Fig. 3), electrolytes show superior thermal stability due to the formation of ion pairs among solvents and Li^+^, which prevent the same type of molecules to cluster at lower temperatures. Likewise, our results show that the EC fraction, in addition to the second solvent in the binary mixture, has also a direct influence on the thermal stability. LP30 (Fig. 4, A to C) and LP40 (Fig. 4, D to F), both with a molar fraction above 0.56, solidify as a mixture below their transition temperatures [−21.9°C for LP30 and −18.1°C for LP40; (*38*)]. However, both present the characteristic behavior attributed to the second solvent (EC-X for 100-X:X fraction, where X is either DMC or DEC) in the mixture in the first cycle to LT. The DMC in LP30, having a higher transition temperature, causes the diffusion to decrease slowly until the sample is fully solidified. The DEC in LP40, with a lower transition temperature, maintains a decreasing slope indicating not a phase transition but a supercooled state.

In Fig. 4 (A to C) at −23°C, it is demonstrated that, although solidification of electrolytes without composition changes is difficult to identify visually, the quantitative nature of the α_rel_ is capable of detecting those changes. For instance, while the α_rel_ change can be measured, the solid state of LP30 at −23°C can be visually confirmed only by analyzing the bubbles formed in the cuvette at −15°C (Fig. 4B), which is characteristic of a transition from solid to liquid when a confined solution experiences supercooling (*39*). Furthermore, the transition induces a rearrangement of the solvents at the top and bottom of the cuvette at −5°C, indicating a change of concentration and, again, a phase transition of the bottom region modifying the transition temperature (bottom region from −5° to 5°C). This change is similar to that of EC-DMC in Fig. 3A, especially at HTs (>20°C), where the α_rel_ in the top and bottom regions remain separated but constant. Once a separation occurs in the electrolyte mixture, exposure to the temperatures below their freezing point will increase the level of separation, create agglomerates, and shift the transition temperature as seen in the last cuvette at −23°C (Fig. 4B, green rectangle). Similar behavior is observed for the LP40 electrolyte (Fig. 4, D to F), where the first solidification is identified in the *T*_img_ and the *r*α_img_, but a change in the α_rel_ at −15°C indicates the phase change. The mixture then remains separated at a constant ratio during the next temperatures of the first cycle to HT, until a clear separation at 5°C, which leads to two defined phases at −23°C of the second cycle to LT.

The low concentration of EC and the lithiated salt improved the thermal stability of LP47 as shown in Fig. 4 (G to I), where no phase transition nor the separation of solvents is observed in the tested temperature range. Our results in Figs. 3 and 4 demonstrate that, after a freezing event, molecules reorganize preferentially to molecules of their own kind (similar diffusion, concentration, and transition temperature). The likelihood of mixture separation increases with the concentration of molecules with greater transition temperature and the difference between diffusivities of constituent molecules. Understanding the limitations of the combination of cyclic and linear molecule solvents in new mixtures is essential for the stable and safe performance of batteries, particularly during fast charging with constantly increasing C-rates.

### Temperature-dependent performance in Li-Po commercial batteries

**Fig. 5.**
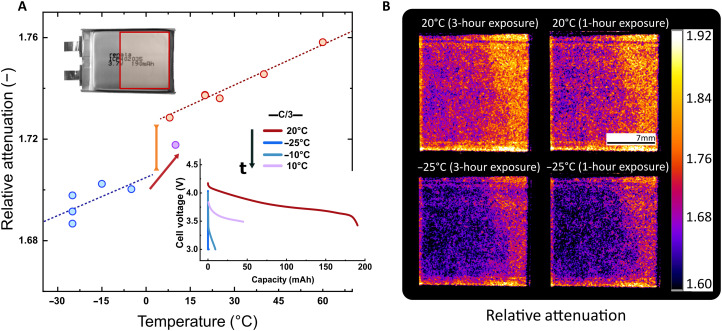
Stability of a commercial Li-Po battery studied via the α_rel_. (**A**) Commercial Li-Po α_rel_ values at the given temperature. The red dashed line is the α_rel_ slope for temperatures above the transition temperature (previous to any phase change). The blue dashed line is the α_rel_ slope after the electrolyte solidification. The purple data point at 10°C was taken after the −5°C one (red arrow) and denotes the transition from solid to the original gel-like aggregation state of the electrolyte. The orange line emphasizes the change of the α_rel_ and the phase change between 8° and −5°C. The capacity curves were measured at C/3 rate for a specific temperature and time (*t*) denoted by the black arrow. (**B**) The *r*α_img_ of the Li-Po battery at two exposures in sequential order: first 20° and −25°C for 3-hour exposure and second both temperatures with 1-hour exposure. Regions with lower α_rel_ (purple) indicate a higher concentration of the gel-like electrolyte. This inhomogeneity does not affect the battery performance. The difference in exposures yields a statistical error below 0.6% for a 99% confidence interval.

Batteries are complex electrochemical devices where a limited amount of electrolyte interacts with internal materials. In our study, we analyzed a commercial lithium polymer (Li-Po) battery from Renata (ICP402035) at various temperatures and exposure times (3 hours and 1 hour) to demonstrate the detection of phase changes in battery electrolytes and to quantify the statistical variations obtained when reducing exposure time, respectively. Because this battery is a commercial device, we were unable to obtain an empty cell without electrolyte (Ref) for the referencing step described in the “Image processing” section. Therefore, the α_rel_ values shown in Fig. 5 correspond to all materials present in the system (electrodes, electrolyte, separator, current collectors, and casing). The electrolyte in this type of battery consists of a combination of dry porous solid polymer and gel-like solvents, to improve thermal stability, conductivity, and ion transfer in the conductive membrane during operation. The battery is rated for a normal constant charge at 0.5C between 0° to 45°C and a normal constant discharge at 1C between −20 to 60°C.

The operando experiment used the same temperature-controlled setup as with the ex situ experiments. However, the sample holder was changed to an aluminum piece of 1 cm, which is outside the field of view (FoV) and provides support for the battery. Because of the small size of the battery (20.5 mm by 36 mm by 4.5 mm) and a stabilization time of 30 min in positive temperatures and 1 hour in negative temperatures, we assume that the set temperature is the same throughout the battery. With these considerations, the changes in the α_rel_ in Fig. 5A represent the overall temperature-dependent change in the cross sections due to changes in the molecular dynamics of the battery materials, more specifically in the gel-like electrolyte due to its H content. As demonstrated in the ex situ experiments, samples without a phase transition (liquid to solid or vice versa) only display a change in the α_rel_ proportional to the change in temperature. When a phase transition occurs, the α_rel_ experiences a steep decrease (between −5° and 8°C; Fig. 5A), and the constant temperature-dependent trend continues. The blue points in the plot indicate a solid state of the electrolyte, which correlates to the substantial decrease in conductivity in the operando battery data to the point where the battery no longer performs (Fig. 5A, inset) at −23°C. Increasing the temperature to −10°C slightly makes the battery perform at approximately 5% of its original capacity. It is only at the next positive temperature (10°C) that the battery recovers and surpasses a 25% capacity rate with stable performance. The capacity curve at 10°C contains only the information from the first hour of discharge at C/3 due to a failure in the connection between the potentiostat and the battery. Nevertheless, the impact of LT on battery performance is evident, with an estimated 22% decrease in maximum capacity compared to its commercial expected rating between 20° and 10°C.

After the electrolyte solidification (temperatures above 10°C), the return of the α_rel_ to the original constant temperature-dependent slope indicates that only physical changes, i.e., phase transition without chemical modifications, took place when exposing the battery to temperatures below its application rate. These findings emphasize the importance of considering the thermal history when calculating the temperature limitations of new battery electrolytes. The α_rel_ obtained with ToF-NI effectively elucidates these temperature-dependent changes. To assess the damage in the battery resulting from electrolyte solidification, postmortem x-ray and spectroscopic techniques are recommended. However, the scope of this investigation focuses on the detection of physical and chemical changes in operando batteries.

In this experiment, we reduced the exposure per acquisition to 2 min per ToF as a result of the higher flux at the Imaging with Cold Neutrons (ICON) compared to the BOA beamline. Likewise, we tested 3 and 1 hour of exposure (Fig. 5B) to evaluate the deviation among our measurements, and we conclude that accurate measurements (>1% statistical deviation) of hydrogenous materials require 10 min at the ICON and 30 min at the BOA for ROIs of a minimum of 40 × 40 pixels. By using beamlines with higher flux (such as ODIN at the ESS), the exposure time can be reduced further to improve the acquisition rate and accuracy of measurements. Therefore, we demonstrate the ToF-NI method and analysis of the α_rel_ to be effective tools for studying thermal limitations in battery systems quantitatively and qualitatively to pave the way to viable operando studies in various environmental conditions for portable devices and the transport industry.

### Prospects opened by ToF-NI analysis of battery electrolytes

In summary, we propose a unique approach to image phase and composition changes of electrolytes in batteries via ToF-NI. By using the relative attenuation (α_rel_), we discerned physical and chemical changes (phase transition, concentration, and transition temperature) due to temperature variations and reduce the exposure time required by spectral imaging methods. Above, we demonstrate that the α_rel_ and the relative attenuation transmission images (*r*α_img_) enable fast measurements of battery electrolytes at high accuracy and repeatability for both visual and analytical inspection in a nondestructive manner. New pulsed beamlines with higher flux, i.e., ODIN at the ESS, or better chopper systems in continuous sources can further reduce the exposure time needed and improve the statistics shown in this work. Reducing the exposure is critical for studying materials in general with neutron techniques but especially in the battery field to understand temperature effects and the internal impact of fast charging.

The battery operation at LTs can be improved by limiting EC concentration in the electrolyte mixture. Low EC concentrations prevent molecular agglomeration and separation resulting in early solidification. The overall performance of new electrolyte mixtures and additives is further analyzed via the ToF-NI method, because the physical state of the electrolyte measured using the α_rel_ is correlated to its thermal capabilities and efficiency. Our future work will include a development of Li-ion battery simulation to predict the thermal management, which can be validated by spectral NI results as presented in this study.

ToF-NI is a flexible technique not limited to battery materials but hydrogenous substances in general. Its spatially resolved and nondestructive nature is ideal to investigate electrochemical systems, i.e., fuel cells and batteries, in a broad range of applications to understand temperature implications on material degradation, phase changes, distribution of impurities, and issues related to mass and ionic transport mechanisms. This method is not limited to modified systems requiring optimal access or the extraction of the electrolyte. ToF-NI is an in situ method fully applicable to conventional battery systems including cylindrical, prismatic, and coin cells. The only limiting factor is the battery thickness due to the boundary conditions of the Beer-Lambert law, i.e., very thin or thick batteries with neutron transmission close to 1 or 0, respectively. It can be used to visualize electrolyte solidification at LTs or temperature distribution during fast charging. In consequence, this method will bring key insights to help resolve the limitations of Li-ion batteries in terms of fast charging and LT operation.

## MATERIALS AND METHODS

### Experimental design

The results presented in this investigation consisted of experiments performed at three beamlines of two neutron facilities: a continuous spallation source [ICON (*40*) and BOA (*41*, *42*) in the Swiss Spallation Neutron Source (SINQ)] at the Paul Scherrer Institut (PSI) in Switzerland and a pulsed spallation source [IMAT (*43*) in ISIS] at the Rutherford Appleton Laboratories in the United Kingdom. These beamlines offer the use of ToF-NI method by installing a chopper disk without modifications to the temperature-dependent setup and ToF distance. The beamlines and neutron sources have unique characteristics. BOA and ICON are cold neutrons beamlines with a flux of ~2.5 × 10^7^ neutrons cm^−2^ s^−1^ and ~5 × 10^7^ neutrons cm^−2^ s^−1^, respectively. Both setups in the SINQ used a 40-mm pinhole and a chopper system that consisted of a Cadmium disk (300-mm diameter) with a 20% duty cycle at 88 Hz, corresponding to a wavelength range of 1.1 to 7.9 Å. The chopper-to-detector distance (flight path) was 550 ± 5 cm, and the sample-to-detector distance was 5.63 ± 0.5 cm (accounting for the complete temperature setup and sample holder thicknesses). On the other hand, the IMAT is a cold neutrons beamline with a flux of ~3.6 × 10^7^ neutrons cm^−2^ s^−1^ (open position), where neutrons are produced by a pulsed spallation source at two repetition rates of 10 Hz (single-frame width) and 5 Hz (double-frame width) for wavelength ranges of ~0.68 to 6.8 Å and ~2 to 14 Å, respectively. The full aperture (100-mm diameter) was used for both repetition rates. The flight path was 560 ± 5 cm, and the sample-to-detector distance is the same as that of BOA and ICON for the 5-Hz repetition rate and 0.8 ± 0.05 cm at 10 Hz (without temperature setup).

A microchannel plate (MCP) detector (*44*) with a 28 mm–by–28 mm FoV and 55-μm pixel size was used in normal transmission geometry. An aluminum enclosure was modified in-house to hold and isolate a suspended aluminum heat exchanger plate, electronics, thermocouples, and a refrigerant circuit. The temperature-controlled setup pumped refrigerant (60/40 ethylene glycol-water mixture) from the circulator (FP51-SL refrigerated circulator, JULABO GmbH) to the aluminum plate inside the enclosure in a loop through isolated 6-mm stainless steel pipes, where the heat was exchanged via conduction between the sample and the plate. The temperature was controlled remotely with six thermocouples inside the housing: four dedicated to the sample holder and two on the bottom and top walls of the box. The latter two thermocouples controlled four heat bands (two bottom and two top) that maintained the surface of the housing above the dew point (at the time of the experiment) to avoid condensation. All electronics were controlled with LabView and NICOS software in the temperature-controlled setup and beamlines, respectively. The deviations among set points and temperatures were within ±0.5°C; 30-min stabilization was used for temperatures above −10°C, 2 hours otherwise.

To measure the transmission of organic solvents, organic binary mixtures, and electrolytes, we used an aluminum sampler holder with 16 cuvettes of 3-mm thickness to optimize the limited FoV in the detector. Four samples can be measured in a single batch at the same time. In the BOA beamline, the transmission of battery electrolytes and components was measured in temperature ramps with a fixed exposure of 30 min with 5-min ToF acquisition cycles.

1. The first cycle to LT: from 20°C down to −23°C in steps of 10°C. The last point, from −10° to −23°C, with a Δ-step of 13°C, as it is the minimum reached with this setup.

2. The first cycle to HT: from −23°C up to 51°C and back to 20°C in steps of 10°C. The first point, from −23° to −15°C, with a Δ-step of 8°C for an effective Δ-step of 5°C from LT to HT. The specific point at 17°C was taken for reproducing reference measurements (*23*).

3. The second cycle to LT: from 20°C down to −23°C in steps of 10°C. The first point, from 20° to 5°C, with a Δ-step of 15°C for an effective Δ-step of 5°C from HT to LT.

Similarly, at IMAT, we measured the transmission of battery electrolytes and components. For the 5-Hz repetition rate, we measured only the first cycle to LT from 20° to −22°C in steps of 10°C and then 20°, 60°, and 20°C single temperatures as a control and to compare the σ*_T_*(λ) deviation given the source and chopper setups for the ToF-NI method. The total exposure time was fixed to 1 hour with 5-min ToF acquisition cycles. This work only used the IMAT variation of 10 Hz (*23*) for reproducibility and control of the σ*_T_*(λ) at long exposures (4-hour full wavelength range).

The in situ measurement took place at the ICON beamline. We used a Renata commercial Li-Po battery (described below) in a temperature range of −25° to 60°C with an effective step of 10 ± 2°C. To understand the influence of total exposure time in commercial products, we used two different exposures with 2-min ToF acquisition cycles: 3 hours and 1 hour for both HT and LT. The standard point (20°C) was measured twice, at the beginning and the end of the experiment, with both exposures. The purpose of separating the total exposure time into shorter acquisitions is to prevent acute beam fluctuations, i.e., beam downtimes, from affecting the overall statistics of the measurements. A SP300 potentiostat and EC-Lab software from BioLogic was used to cycle the battery.

### Samples

The samples measured in the holder consist of pure organic solvents from BASF and Sigma-Aldrich: EC, DMC, and DEC. Organic binary mixtures: EC-DMC of 1:1 v/v, EC-DEC of 1:1 v/v, and EC-DEC of 3:7 v/v. Electrolytes: LP30 (1 M LiPF_6_ in EC-DMC of 1:1 v/v), LP40 (1 M LiPF_6_ in EC-DEC of 1:1 v/v), LP47 (1 M LiPF_6_ in EC-DEC of 3:7 w/w). The in situ experiments used a commercial Li-Po battery from Renata (ICP402035) with a capacity of 195 mAh, a voltage of 3.7 V, and a thickness of 4.4 mm.

### Time-of-flight neutron imaging

ToF-NI is a wavelength-resolved technique that requires the use of pulsed sources or chopper disks to facilitate the spread of neutrons in time according to their energy (*E*) or wavelength (λ) as given by the de Broglie relation, $\lambda =h/\sqrt{2m_{n}E}$ (*45*), where *h* is Planck’s constant and *m*_n_ is the neutron mass. The separation prompts a wavelength-dependent interaction with matter that arrives to the detector at a fixed time for a fixed flight path, resulting in wavelength-dependent transmission frames per acquisition over a fixed exposure time. The Beer-Lambert law, *T* = *e*^−Σδ^, describes the effective transmission, *T* = *I*/*I_o_*, in terms of the path length or thickness, δ, and the attenuation coefficient, Σ, of the material, which relates to the number density, *N_i_*, of nuclei in the sample, and the neutron total cross section σ*_T_*(λ). Calculating the σ*_T_*(λ) for nonchanging concentrations and thicknesses, i.e., PE, H_2_O, EC, DMC, and DEC, requires only to apply Beer-Lambert law to the transmission given by the ToF-NI method, as shown in Fig. 1A. However, complex systems such as binary mixtures and electrolytes may change their concentration ratio due to partial solidification when subjected to extreme temperatures (*23*), resulting in a shift of the σ*_T_*(λ) (Fig. 2B). To investigate these temperature-dependent physical and chemical changes, it is practical to use the relative attenuation, α_rel_, which is the ratio between the total cross section at LWs, σ*_T_*(λ_LW_), and the total cross sections at SWs, σ*_T_*(λ_SW_). Thus, α_rel_ = σ*_T_*(λ_LW_)/σ*_T_*(λ_SW_). The α_rel_ is useful particularly when analyzing the thermal history of hydrogenous materials, because physicochemical changes reflect mainly in the σ*_T_*(λ_LW_), resulting in phase-dependent charts (Fig. 1C), while providing spatially resolved information (Fig. 1D). The grayscale transmission images, *T*_img_, constitute the effective transmission after the image processing, and the relative attenuation images, *r*α_img_, correspond to the calculated two-dimensional (2D) α_rel_ in the postprocessing step after the selection of σ*_T_*(λ_SW_) and σ*_T_*(λ_LW_) regions. The parameters governing ToF-NI, e.g., spatial resolution, exposure time, chopper systems, and duty cycle, depend on the source (flux and beam profile) and the detector settings (described above).

### Image processing

All experiments used an MCP detector, which generates a fixed number of ToF frames per acquisition: in BOA, 109 frames per cycle (12 ms), averaged over 5-min ToF exposure; in ICON, 109 frames per cycle (12 ms), averaged over 2-min ToF exposure; and in IMAT, 456 and 1090 frames per cycle, averaged over 5-min ToF exposure at 10 and 5 Hz, respectively. The baseline of the processing framework is divided into three steps.

1. Preprocessing: This consists of algorithms (sorting and event overlap correction) required for documentation purposes and to perform corrections intrinsic to the detector software; operations in this step do not influence the result. Sorting categorizes acquisitions into batches according to the sample and input temperature from a metadata file generated during the experiments; the folder arrangement does not affect the thermal history. Event overlap correction is an exclusive feature of a MCP detector (*46*). The purpose of this step is to reconstruct the timing signal of the periodic flux (acquired by the ToF method) for high input fluxes per acquisition. In this step, only the 5-Hz acquisitions were binned by a factor of 10 (resulting in 109 frames) due to current memory and processing capabilities.

2. Processing: This consists of algorithms [averaging; scrubbing correction, filtering, and background scattering (SBKG) correction; intensity correction; and referencing] required to obtain the effective transmission of a sample. This step is setup specific, and the operations directly affect the result. Averaging calculates the arithmetic mean over all acquisitions belonging to a single temperature, i.e., reducing six 5-min ToF acquisitions of 109 frames (30-min exposure time) into a single averaged acquisition. Scrubbing correction removes the time and flux-dependent loss of counts (*47*) by interpolating open-beam (OB) ToF images taken before and after each experiment. The code assigns a weight (from 0 to 1) to each OB according to the time that it was taken from the sample; the ratio is calculated thereafter. Filtering removes zero (dead) pixels with an outlier remover (threshold set to zero) and applies a 2D median filter with a kernel size of three from the SciPy signal library to remove white spots. The SBKG correction removes the secondary scattered neutrons by computing 2D images through the interpolation of regions among 10-mm-thick Boral black body (BB) stripes at the bottom, middle, and top of the FoV. Although the MCP detector is not substantially affected by SBKG, resulting SBKG images are subtracted frame-wise from the measured transmission (*23*) to improve data accuracy. Intensity correction (*1*) accounts for the beam intensity fluctuations during the experiment. The area between the middle and top of the BBs holds no sample and was defined as a nonchanging region (NCA) (OB within the same FoV as the sample). The ratio between NCA and the reference (Ref) (empty sample holder including all parts of the setup in the FoV) adjusts the intensity of each frame. Referencing removes the contribution of all parts in the setup, i.e., enclosure, heating plate, and sample holder, by dividing the target sample with the Ref ($T=\frac{I}{I_{o}}=\frac{\mathrm{sample}}{\mathrm{Ref}}$). The result of the processing is sets of relative transmission images with constant wavelength efficiency for each sample and temperature.

3. Postprocessing: This consists of algorithms (cross-sectional calculation and relative attenuation calculation) required for visualizing, extracting, and analyzing the effective transmission; operations in this step convert pixel values into physical variables. Cross-sectional calculation computes the σ*_T_*(λ) of each sample (ROI-wise) with Beer-Lambert law given the constants described above. Relative attenuation calculation computes the α_rel_ (image-wise) for specific SW and LW regions. These regions may vary depending on the experiment setup, but, generally, they are kept between 1.0 and 3.5 Å and between 5.5 and 9.0 Å for SW and LW, respectively (*23*). This work focuses on the capability of α_rel_ to follow physicochemical and temperature-dependent changes. Therefore, we only use the σ*_T_*(λ) for the calculations in Fig. 1 and to perform the statistical analysis (section below) for every source. All algorithms presented here were developed in Python within a general ToF-NI framework with an MCP detector and were implemented in Jupyter Notebook. The *r*α_img_ presented in the figures have a median filter with a 1-pixel radius (ImageJ software) for visualization purposes. However, the α_rel_ data points are measured without a filter. The color lookup tables used only for visualization purposes for *T*_img_ and *r*α_img_ are inverse grayscale and fire, respectively, from the ImageJ software.

### Statistical analysis

We calculated the statistical variation using a one-sided 99% confidence interval with Student’s *t* distribution to evaluate the σ*_T_*(λ) margin of error [$t(n-1)/\sqrt{n}$], where *n* is the number of acquisitions for a fixed exposure time. We performed two evaluations for all experiments: over the entire σ*_T_*(λ) spectrum of each beamline and source and over the SW and LW regions used to compute the α_rel_. The difference is that the σ*_T_*(λ) spectrum includes regions affected by the pulse overlap (*18*) (only BOA and ICON) not present in the α_rel_, which provides an idea of the true accuracy, even for short exposure times, for future reference. At the BOA (5-min ToF with 30-min total exposure time), we estimated a 2.14% of variation for the entire (1.1 to 7.9 Å) σ*_T_*(λ) spectrum and 1.23% of the SW and LW regions in the α_rel_ range. At the IMAT, we estimated a 0.27% of variation with the 10-Hz repetition rate (5-min ToF with 3 hours total exposure time) and a 0.47% of variation with the 5-Hz repetition rate (5-min ToF with 1 hour total exposure time) for a σ*_T_*(λ) range of 0.7 to 9.7 Å and 1.1 to 10.0 Å, respectively. During the in situ experiments at the ICON (2-min ToF acquisitions), the 3- and 1-hour exposures yielded a 0.62 and 0.67% of variation for the 1.1- to 7.9-Å σ*_T_*(λ) spectrum, respectively; and 0.56 and 0.62% of variation over the α_rel_ SW and LW regions, respectively. Similarly, for the α_rel_ results, we evaluated the statistical variation of the ROI size (one-sided 99% confidence interval with Student’s *t* distribution) for 25 arbitrarily selected regions over four different sizes (10 × 10, 20 × 20, 30 × 30, and 40 × 40 pixels) within three samples of different natures at 20°C: a pure hydrogenous material (H_2_O), an organic binary mixture (EC-DMC of 1:1 v/v), and an electrolyte (LP30). The variation for all ROI sizes over the population is 0.96 ± 0.11%, 0.58 ± 0.09%, 0.37 ± 0.08%, and 0.26 ± 0.04%, respectively. Therefore, the results presented in this work are produced with ROIs of at least 40 × 40 pixels. The statistical variations among our measurements and literature references (for H_2_O and PE) (*26*, *27*) are below 2%.

Our statistical analysis confirms that the main source of deviation in our measurements, compared to the literature and for ToF-NI experiments with a MCP detector, derives from systematic errors, especially in continuous sources with single chopper systems such as SINQ (SW region in Fig. 1B). A high duty cycle in a chopper yields a higher flux for the ToF method reducing the exposure time required. However, it comes at an expense of wavelength resolution and pulse overlap. Although pulse overlapping reduces the accuracy of the σ*_T_*(λ) calculation for wavelengths below 3 Ȧ, the α_rel_ remains unaffected because the overlap is a feature of the setup (physical interaction between neutrons and chopper), which remains constant throughout the experiments.

We perform an additional analysis of the number of OB acquisitions required between the experiments to account for the scrubbing and to reduce the overall experiment time. We measured the difference for single, three, and six acquisitions (both 5-min and 2-min ToF) and determined that a 5-min exposure (one 5 min or three 2 min) is sufficient to consider the loss of counts. Our experiments produced less than 0.15 and 1.75% of scrubbing over the 28 mm–by–28 mm FoV with a maximum exposure of 90 min (three batches of 30-min total exposure) and 3 hours at BOA and ICON, respectively. The scrubbing is setup dependent, and the change of contrast presented in this analysis should only be considered for similar setups.
